# Malnutrition and cerebral intraparenchymal damage in patients with thrombosis of dural sinuses and/or cerebral veins

**DOI:** 10.1186/s12883-023-03491-1

**Published:** 2023-12-20

**Authors:** Weiwei Xiang, Yangyang Liang, Zhibo Chen, Hanmin Wang, Yangtai Guan, Dewei Xie

**Affiliations:** 1 Department of Neurology, Ren Ji Hospital, Shanghai Jiao Tong University School of Medicine, No.160, Pujian Road, Shanghai, 200127 China; 2 The College of Renji, Wenzhou Medical University, Wenzhou, 325000 Zhejiang China; 3 Department of Neurology, The First Affiliated Hospital of Wenzhou Medical University, Wenzhou, 325000 Zhejiang China

**Keywords:** Malnutrition, CVT, Cerebral intraparenchymal damage, Nomogram

## Abstract

**Backgrounds:**

Thrombosis of dural sinuses and/or cerebral veins (CVT) is an uncommon form of cerebrovascular disease. Malnutrition is common in patients with cerebrovascular disease, and early assessment of malnutrition and individualized nutritional treatment have been reported to improve functional outcomes of these patients. As for CVT patients, little is known about whether these patients would suffer from malnutrition. Also, the correlation between malnutrition and cerebral intraparenchymal damage (CID) in CVT patients was rarely studied.

**Methods:**

Patients with CVT were retrospectively included in this observational study. Multivariate logistic regressions were used to investigate the effects of nutritional indexes on the risk of CID. Subsequently, we used the independent risk factors to construct the nomogram model, and the consistency index (C-index), calibration curve and decision curve analysis (DCA) to assess the reliability and applicability of the model.

**Results:**

A total of 165 patients were included in the final analysis. Approximately 72.7% of CVT patients were regarded as malnourished by our malnutrition screening tools, and malnutrition is associated with an increased risk of CID. Prognostic Nutritional Index (PNI) (OR = 0.873; CI: 0.791, 0.963, *p* = 0.007) remained as an independent predictor for CID after adjustment for other risk factors. The nomogram model showed that PNI and gender have a great contribution to prediction. Besides, the nomogram model was consistent with the actual observations of CID risk (C-index = 0.65) and was of clinical significance.

**Conclusions:**

We reported that malnutrition, as indicated by PNI, was associated with a higher incidence of CID in CVT patients. Also, we have constructed a nomogram for predicting the risk of CID in these patients.

## Introduction

 Thrombosis of dural sinuses and/or cerebral veins (CVT) is a cerebrovascular disease caused by cerebral venous reflux obstruction [1, 2]. This disease is an uncommon form of cerebrovascular disease, and the incidence of CVT is up to 1.3–1.6 per 100,000 people [3]. CVT usually affects young and middle-aged individuals and occurs more frequently in females [1, 2]. The high variability in risk factors, clinical manifestation, and neuroimaging of CVT poses challenges to the prognosis [4]. Thus, carrying out risk stratification of CVT patients is of clinical significance.

Malnutrition has been demonstrated to be involved in the clinical outcomes of patients with cerebrovascular disease, such as acute ischemic stroke (AIS) [5–9]. Malnutrition is common in AIS patients, and it can hinder neurological functional recovery, prolong the duration of hospital stays and increase the mortality rate in these patients [5–7]. Of these patients, early assessment of malnutrition and individualized nutritional treatment have been reported to improve functional outcomes [10]. As for CVT patients, little is known about whether these patients would suffer from malnutrition. Patients with cerebrovascular disease have a high incidence of cerebral intraparenchymal damage (CID) and it poses great threats to the neurological functional recovery of these patients [11]. Up until now, the correlation between malnutrition and CID in CVT patients was rarely studied.

Therefore, this study sought to use the following nutritional screening tools: The Controlling Nutritional Status (CONUT) score the Nutritional Risk Index (NRI), and the Prognostic Nutritional Index (PNI) to screen the risk of malnutrition in CVT patients. We also aimed to evaluate associations between nutritional status and the risk of cerebral intraparenchymal damage (CID). Besides, we constructed a nomogram to help neurologists stratify patients and cope with modifiable risk factors.

## Methods

### Patient enrollment

This retrospective study was performed in the first affiliated hospital of Wenzhou Medical University. Acute/subacute CVT patients were enrolled consecutively between 2016 and 2022. The diagnosis of CVT was in accordance with international standards [12, 13]. The inclusion criteria were as follows: (1) patient more than 16 years old; (2) patients without severe hepatic or renal diseases; (3) available valid information on clinical data and functional outcome.

### Data collection

Demographics (age and gender), medical history (smoking, drinking, hypertension, diabetes mellitus, onset to treat, perinatal period in females and previous Infections), and laboratory parameters (total cholesterol, triglyceride, high-density lipoprotein cholesterol, low-density lipoprotein cholesterol, albumin, white blood cell, Neutrophil, Lymphocyte, homocysteine and D2 polymers) were gathered and analyzed. The symptoms and signs (headache, focal neurologic deficit, seizure, conscious disturbance) and location of thrombus (sigmoid sinuses, transverse sinuses, straight sinus, superior sagittal sinus, deep CVT, cortical vein) were also recorded. CID was defined as intracerebral hemorrhage or cerebral infarction with imaging manifestation (CT/MRI) and clinical manifestations. Modified Rankin Scale (mRS) was assessed over the phone after 3 months of discharge.

Body mass index (BMI) was calculated using the following formula: [weight (kg)]/[height (m)]^2^. The CONUT score was scored according to the levels of serum albumin concentration, lymphocyte count and total cholesterol. The specific scoring system was as follows: albumin concentration ≥ 35.0 (g/L), 0 point; 30.0–34.9 (g/L), 2 points; 25.0–29.9 (g/L), 4 points; and < 25.0 (g/L), 6 points; lymphocytes count ≥ 1.60 (10^9^/L), 0 point; 1.20–1.59 (10^9^/L), 1 point; 0.80–1.19 (10^9^/L), 2 points; and < 0.8 (10^9^/L), 3 points; and total cholesterol ≥ 180.00 (mg/dL), 0 point; 140.00–179.99 (mg/dL), 1 point; 100.00–139.99 (mg/dL), 2 points; and < 100.00 (mg/dL), 3 points. The total score was calculated and divided into 4 levels: normal (0 to 1), mild malnutrition (2 to 4), moderate malnutrition (5 to 8) and severe malnutrition (9 to 12). The NRI is calculated as 1.489 × serum albumin (g/l) + 41.7 × (current body weight in kg/ideal body weight in kg) [14]. The NRI was classified as severe malnutrition (< 83.50), moderate malnutrition (83.50–97.49), mild malnutrition (97.50–99.99), and normal (≥ 100). The PNI score was calculated using 10 × serum albumin (g/dl) + 0.005 × total lymphocyte count (mm^3^) and then divided into 3 groups: severe malnutrition (< 35), moderate malnutrition and normal (> 38).

### Statistical analysis

Continuous variables were checked for normality using the Kolmogorov–Smirnov test. These variables were expressed as the me an ± standard deviation (SD) or median with interquartile range (IQR). Student’s t-test or Mann-Whitney U test was used to test the intergroup difference of continuous variables, whichever is appropriate. Categorical variables were expressed as numbers and percentages, and the χ^2^ test or Fisher exact test was used to test the intergroup difference of these variables. Multivariate logistic regression analysis was used to examine the associations between each of the potential risk factors and CID. The nomogram model was constructed using variables with a *p-*value less than 0.05 in multivariate regression analyses, and was compiled with R software. A *p*-value less than 0.05 was considered significant. All statistical analyses were performed using SPSS 24.0.

## Results

### Basic characteristics

A total of 165 patients with a median age of 42.0 years were included in the final analysis (Table 1). Male patients accounted for 57.6% of total patients. More details on medical history, laboratory findings, and clinical characteristics were illustrated in Table 1.

**Table 1:** Baseline characteristics of the participants with or without CID

Characteristics	Total *n* = 165	CID, *n* = 87	Non- CID, *n* = 78	*p*
**Demographics**				
Age (year), median (IQR)	42.0 (19.5)	43.0 (18.0)	42.0 (22.25)	0.747
Female, n (%)	70 (42.4)	46 (52.9)	24 (30.8)	**0.004**
**Medical history**				
Smoking, n (%)	56 (33.9)	28 (32.2)	28 (35.9)	0.615
Drinking, n (%)	44 (26.7)	23 (26.4)	21 (26.9)	0.944
Hypertension, n (%)	40 (24.2)	23 (26.4)	17 (21.8)	0.487
Diabetes mellitus, n (%)	15 (9.1)	8 (9.2)	7 (9.0)	0.961
Onset to treat, median (IQR)	6.0 (7.5)	5.0 (7.0)	7 (10.5)	0.140
Perinatal period in females, n (%)	14 (20.0)	11 (23.9)	3 (12.5)	0.413
Previous Infections, n (%)	11 (6.7)	7 (8.0)	4 (5.1)	0.453
**Laboratory findings**				
TC (mmol/L) median, (IQR)	4.71 (1.46)	4.61 (1.24)	4.78 (1.53)	0.176
TG (mmol/L), median (IQR)	1.45 (0.76)	1.36 (0.76)	1.67 (1.00)	**0.004**
HDL-C (mmol/L), median (IQR)	1.02 (0.30)	1.05 (0.32)	0.99 (0.28)	0.123
LDL-C (mmol/L), median (IQR)	2.66 (0.97)	2.64 (1.04)	2.76 (1.07)	0.073
Albumin (g/L), median (IQR)	38.1 (5.40)	37.40 (5.40)	38.75 (4.85)	0.095
white blood cell (10^9^ /L), median (IQR)	8.46 (4.15)	7.90 (3.73)	8.84 (4.69)	0.447
Neutrophil, median (IQR)	6.00 (4.43)	5.80 (4.42)	6.06 (4.75)	0.944
Lymphocyte, median (IQR)	1.54 (1.10)	1.38 (0.95)	1.78 (1.10)	**0.003**
Homocysteine, median (IQR)	13.0 (4.0)	12.0 (5.0)	13.5 (4.0)	**0.024**
D2 polymers, median (IQR)	1.35 (1.58)	1.51 (1.85)	1.08 (1.21)	**0.005**
**Symptoms and signs**				
Headache, n (%)	148 (89.7)	76 (87.4)	72 (92.3)	0.296
focal neurologic deficit, n (%)	76 (46.1)	58 (76.3)	18 (23.7)	**<0.001**
Seizure, n (%)	43 (26.1)	37 (42.5)	6 (7.7)	**<0.001**
Conscious disturbance, n (%)	30 (18.2)	25 (28.7)	5 (6.4)	**<0.001**
**Location of thrombus**				
Sigmoid sinuses, n (%)	100 (60.6)	46 (52.9)	54 (69.2)	**0.032**
Transverse sinuses, n (%)	113 (68.5)	56 (64.4)	57 (73.1)	0.229
Straight sinus, n (%)	31 (18.9)	16 (18.4)	15 (19.2)	0.890
Superior sagittal sinus, n (%)	101 (61.2)	53 (60.9)	48 (61.5)	0.935
Deep CVT, n (%)	9 (5.5)	7 (8.0)	2 (2.6)	0.228
Cortical vein, n (%)	22 (13.3)	15 (17.2)	7 (9.0)	0.119
**Follow-up and functional outcome**				
Recurrence of the disease, n (%)	14 (8.5)	6 (6.90)	8 (10.3)	0.439
Epilepsy, n (%)	10 (6.1)	9 (10.3)	1 (1.3)	**0.035**
mRS score at 3 months, median (IQR)	0 (1.0)	1.0 (2.0)	0 (1.0)	**0.001**
**Nutritional indicators**				
Body mass index (kg/m^2^), SD	23.63 (3.01)	23.22 (2.92)	24.09 (3.05)	0.065
CONUT score, median (IQR)	2.0 (2.0)	2.0 (3.0)	1.5 (3.0)	0.012
NRI, median (IQR)	100.68 (11.37)	100.00 (11.52)	103.31 (10.53)	0.031
PNI, median (IQR)	46.70 (8.70)	44.25 (7.70)	47.70 (7.96)	0.002

*Abbreviations*: *CID *Cerebral intraparenchymal damage, *IQR *Interquartile range, *TC *Total cholesterol, *TG *Triglyceride, *HDL-C *High-density lipoprotein cholesterol, *LDL-C *Low-density lipoprotein cholesterol, *mRS *Modified Rankin Scale, *SD *Standard deviation, *CONUT score *The controlling nutritional status score, *NRI *The Nutritional Risk Index, *PNI *Prognostic nutritional index

### Prevalence of malnutrition in CVT patients

**Fig. 1:**
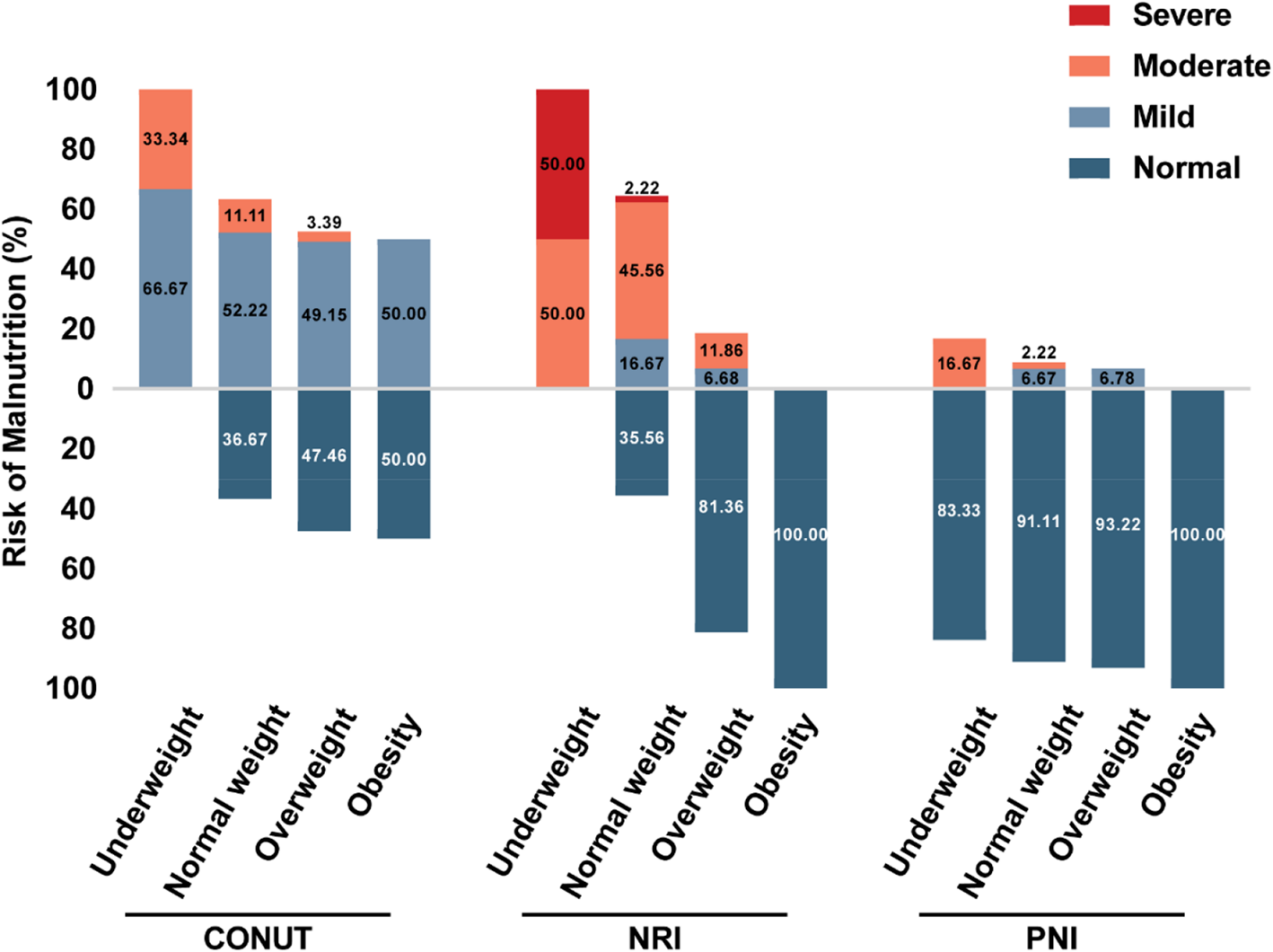
Risk of malnutrition according to malnutrition indexes by subgroups of patients according to BMI. BMI was divided into 4 groups, including underweight, normal weight, overweight and obesity.The percentage of malnutrition was defined according to malnutrition screening tools (CONUT, NRI, and PNI) in different subgroups of patients. Abbreviations: BMI, Body mass index; CONUT score, the controlling nutritional status score; NRI, The Nutritional Risk Index; PNI, prognostic nutritional index

BMI was divided into 4 groups, including underweight (< 18.5), normal weight (18.5–23.9), overweight (24.0–27.9) and obesity (≥ 28.0) [15]. The percentage of malnutrition was defined according to malnutrition screening tools, including PNI, CONUT, and NRI, in underweight, normal weight, overweight and obesity patients. According to these malnutrition screening tools 66 (40.0% for CONUT), 75 (45.5% NRI) and 13 (7.9% for PNI) patients suffered from mild to severe malnutrition (Fig. 1). Of all CVT patients, 27.3% were not malnourished by any index, and 7.3% were regarded as malnourished by all 3 indexes. Malnutrition is common in underweight patients, whether evaluated by CONUT (100.0%), NRI (100%), or PNI (16.7%) scores. Of note, a proportion of overweight and obese patients was also considered malnourished according to our malnutrition screening tools (52.1% for CONUT; 15.9% for NRI; 5.8% for PNI).

### Relationship between malnutrition indexes and CID

**Fig. 2:**
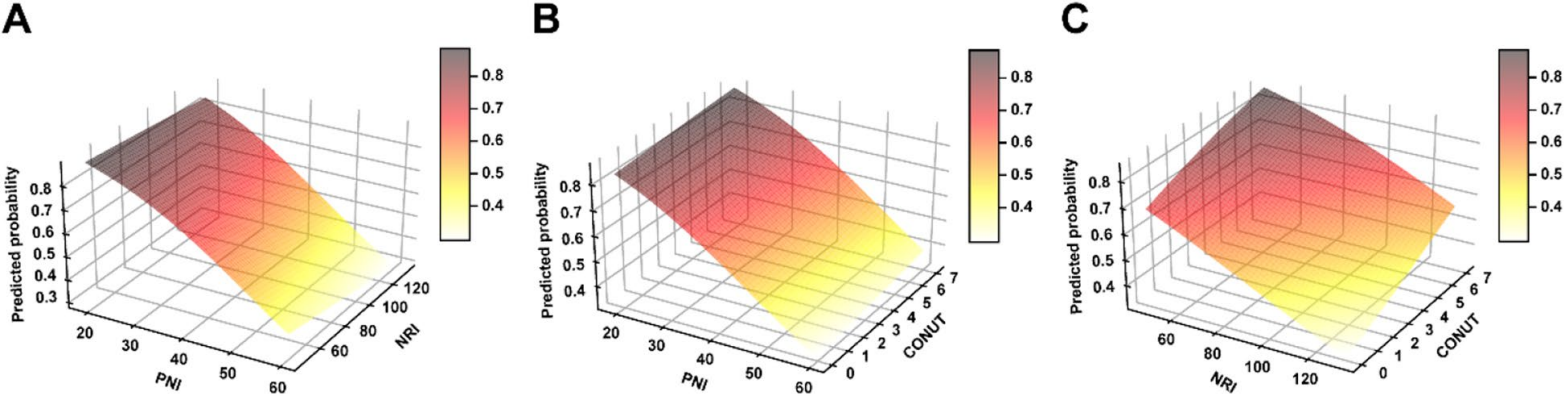
Association between the predicted probability of CID and CONUT, NRI, and PNI. The predicted probability of CID increased as CONUT increased and PNI and NRI decreased.Abbreviations: CID, cerebral intraparenchymal damage; CONUT = controlling nutritional status score; NRI = nutritional risk index; PNI = prognostic nutritional index

Patients with CID had higher CONUT score (*p* = 0.012), and lower NRI (*p* = 0.031) and PNI (*p* = 0.002) scores, when compared with patients without CID. The differences in BMI (23.22 ± 2.92 vs. 24.09 ± 3.05, *p* = 0.065) were borderline significant. Figure 2 demonstrated that the predicted probability of CID increased accordingly as NRI and PNI decreased and CONUT increased. CONUT score [odds ratio (OR) = 1.211; confidence interval (CI): 1.006–1.458] was significantly positively correlated with the risk of CID in unadjusted logistic regressions models, while continuous PNI (OR = 0.931; CI: 0.881, 0.985), NRI (OR = 0.970; CI: 0.937,1.004) and BMI (OR = 0.906; CI: 0.815,1.007) were inversely associated with CID (Table 2). These results indicated that malnutrition was correlated with the occurrence of CID. In multivariate regression analyses, PNI remained significant after adjustment for potential risk factors (OR = 0.873; CI: 0.791, 0.963 for continuous PNI). Furthermore, PNI was included in multivariate regression analyses as categorical variables (divided into two groups via the cutoff value) (OR = 3.591; CI: 1.424–9.058).

**Table 2:** Logistic regressions of the relationships between malnutrition screening tools (PNI, CONUT, NRI, and BMI) and the risk of CID

Malnutrition indexes	Model1 (unadjusted)	Model 2 (adjusted)
	**OR (95%CI)** ***p***	**OR (95%CI)** ***p***
PNI (continuous)	0.931 (0.881, 0.985), *p* = 0.013	0.873 (0.791, 0.963), *p* = 0.007
PNI (categorical)	2.834 (1.466–5.481), *p* = 0.002	3.591(1.424–9.058), *p* = 0.007
CONUT (continuous)	1.211 (1.006–1.458), *p* = 0.043	-
NRI (continuous)	0.970 (0.937,1.004), *p* = 0.084	-
BMI (continuous)	0.906 (0.815,1.007) *p* = 0.068	-

Model1

Age, Female, D2, PNI, CONUT, BMI, NRI, Homocysteine, albumin, TG, LDL, Sigmoid sinuses

*Abbreviations*: *CID *Cerebral intraparenchymal damage, *BMI *Body mass index, *CONUT score *the controlling nutritional status score, *NRI *The Nutritional Risk Index, *PNI *Prognostic nutritional index, *OR *Odds ratio

### Construction, calibration and predictive performance of the nomogram

**Fig. 3:**
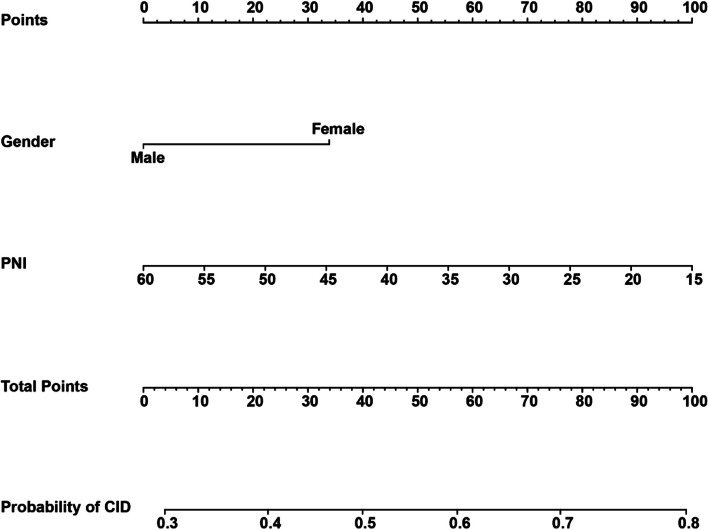
A nomogram predicting the risk of CID in CVT patients. Risk factors, including PNI scores and gender were assigned corresponding scores on the points axis, and the total points was calculated by adding the corresponding scores of all risk factors. The probability of CID in each patient can be obtained through the CID axis relative to total points axis. Abbreviations: CVT, thrombosis of dural sinuses and/or cerebral veins; PNI, prognostic nutritional index; CID, cerebral intraparenchymal damage

According to the results of multivariate regression analyses, we constructed the nomogram which predicted the risk of CID. As illustrated in Fig. 3, PNI scores and gender have a great contribution to the prediction. The PNI scores and gender in the nomogram were assigned corresponding scores on the points axis, and the total points were calculated by adding the corresponding scores of all risk factors. The probability of CID in each patient can be obtained through the CID axis relative to the total points axis.

**Fig. 4:**
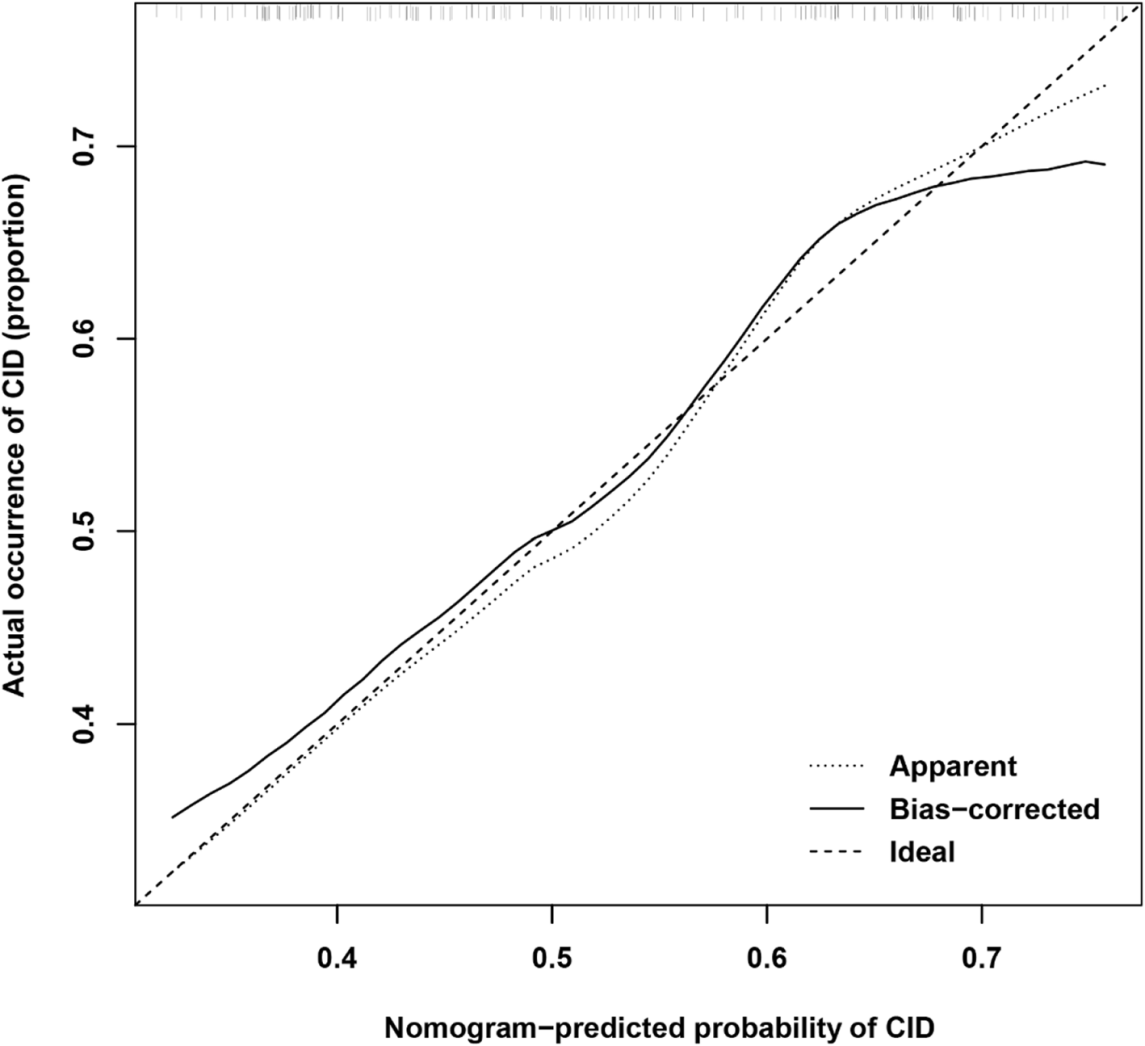
Calibration curves of the nomogram model in the training cohort. The solid line represented the prediction of the nomogram, while the dashed line represented the ideal model for prediction. The closer the two line was, the more accurate the prediction was. Abbreviations: CID, cerebral intraparenchymal damage

**Fig. 5:**
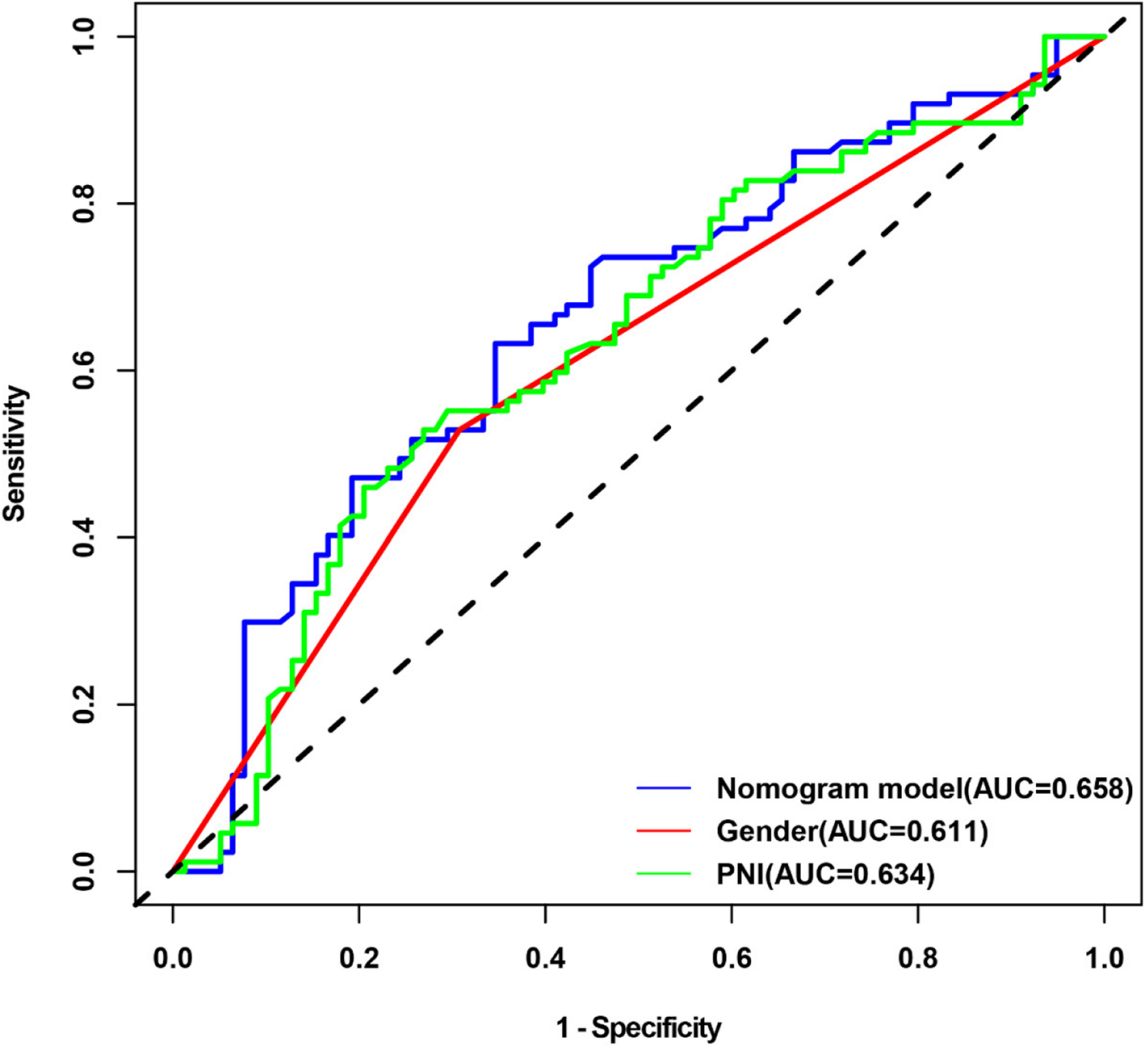
ROC curves of the training cohort. ROC curves illustrated that the AUC of the nomogram model, PNI scores, and gender in the training cohort were 0.658, 0.634, and 0.611, respectively. Abbreviations: ROC, receiver operating characteristic; AUC, area under the curve; CID, cerebral intraparenchymal damage

The calibration curve demonstrated that the nomogram-predicted probability of CID was consistent with the actual probability of CID in the training cohorts (Fig. 4). And the nomogram C-index was 0.65. In addition, The AUC of the nomogram model, PNI scores, and gender were 0.658, 0.634, and 0.611, respectively (Fig. 5).

**Fig. 6:**
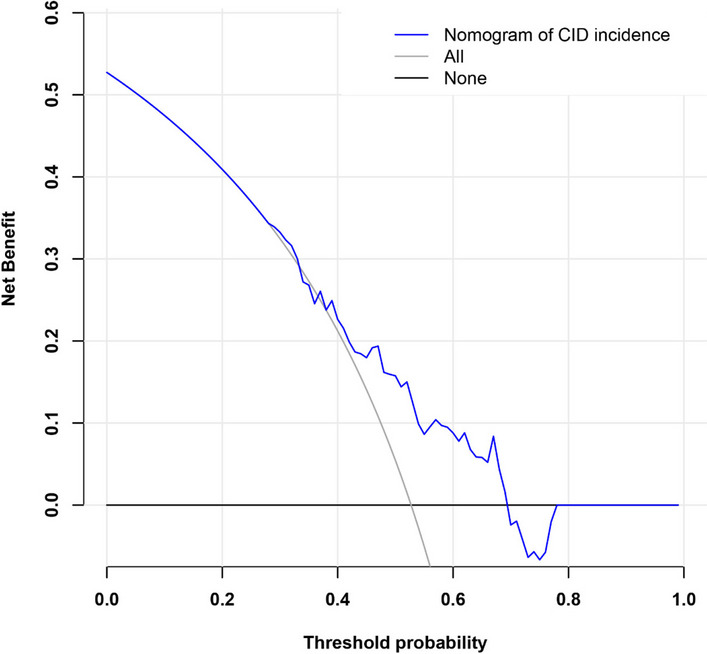
DCA of the nomogram model predicting CID in CVT patients. The solid blue line represented the risk of CID predicted by the nomogram. The grey solid line represented the hypothesis that all patients had CID after CVT. The black solid line represented the assumption that no patients had CID after CVT. Abbreviations: DCA, calibration curve and decision curve analysis; CID, cerebral intraparenchymal damage; CVT, thrombosis of dural sinuses and/or cerebral veins

Predictive performance of nomogram DCA was conducted to assess the clinical significance of the predictive model, which showed if the threshold probability is less than 0.69 and more than 0.29, using this nomogram to predict the risk of CID after CVT adds more benefit than either considering all patients had CID or no patients had CID (Fig. 6).

## Discussion

Our study revealed that CVT patients had a high incidence of malnutrition, and malnutrition is associated with an increased risk of CID. PNI remained as an independent predictor for CID after adjustment for other risk factors, such as age, gender, homocysteine and so on. The nomogram model showed that PNI and gender have a great contribution to prediction. Besides, the nomogram model was consistent with the actual observations of CID risk and was of clinical significance.

Malnutrition is common and important in clinics but is often overlooked by clinicians, which may be due to the exhaustive and time-consuming process of nutritional assessment procedures. Up until now, few studies have evaluated the prevalence of malnutrition in CVT patients [16, 17]. In the present study, by using three easily calculable and widely recognized malnutrition assessment tools, we found that approximately 72.7% of CVT patients were regarded as malnourished by our malnutrition screening tools, and 1.8–33.9% of patients suffered from moderate to severe malnutrition. Malnutrition is common in underweight patients, whether evaluated by CONUT (100.0%), NRI (100%), or PNI scores (16.7%). All three indices consistently suggested a reduced risk of malnutrition as BMI increased. According to previous studies concerning patients with tumors [18–20], PNI was divided into three groups: less than 35 (malnutrition), 35–38 (moderate malnutrition), and greater than 38 (normal). Compared to the patients with tumors, the average value of PNI was higher both in our study and in other studies about cerebrovascular diseases, which was likely to result in a lower proportion of malnutrition in underweight patients and others. Besides, a proportion of obese patients were also malnourished. As malnutrition in CVT patients is often ignored, malnutrition in obese patients is more likely to be overlooked. Obese patients are incapable of effectively utilizing the energy stored in fat in periods of metabolic stress, resulting in the consumption of lean tissue, therefore obese patients may also suffer from malnutrition [21].

CID is relatively common in CVT patients, and it causes devastating consequences in these patients, including epilepsy and neurological. Early recognition of CID is important in optimizing clinical outcomes of CVT patients. Regardless of clinical variables, malnutrition, as indicated by lower PNI, was still correlated to a higher incidence of CID. Consistently, a recent study that reported PNI value was correlated with an adverse 3-month clinical outcome in CVT patients [22]. Therefore, we combine PNI value with gender (another independent risk factor in our multiple regression model) to predict the risk of CID after CVT. The AUC values, C-index and calibration curve indicated the feasibility and prediction ability of the nomogram. And it is of clinical significance to use this nomogram to predict the incidence of CID in CVT patients when the threshold probability of DCA is less than 0.69 and more than 0.29. Therefore, the constructed nomogram can help neurologists stratify patients and cope with modifiable risk factors.

This study is the first to comprehensively examine the associations between malnutrition and the incidence of CID in CVT patients. Besides, we have constructed a nomogram for predicting the risk of CID in these patients. However, there are several limitations in the present study. First, nutritional status was evaluated at a single time point, further dynamic follow-up is needed to comprehensively evaluate the relationship between malnutrition and the risk of CID. Second, this study is an observational study with a relatively small sample size, so further multicenter prospective studies with the addition of a healthy control group are needed to verify our results. Third, the reliability and applicability of this model also need to be confirmed by a multicenter study.

## Conclusion

We reported that malnutrition, as indicated by PNI, was associated with a higher incidence of CID in CVT patients. Also, we have constructed a nomogram for predicting the risk of CID in these patients. The constructed nomogram can help neurologists stratify patients, and further study could focus on whether personalized nutritional intervention will help improve the clinical outcome of CVT patients.

## Data Availability

The data presented in this study are available on request from the corresponding author.
